# Oxidative stress induces meiotic defects of oocytes in a mouse psoriasis model

**DOI:** 10.1038/s41419-022-04948-w

**Published:** 2022-05-19

**Authors:** ZhiQin Zhang, ShouBin Tang, YuYing Jiang, FangYuan Long, Fang He, Jian Liu, ShouYong Gu, Yan Lu, ZhiQiang Yin

**Affiliations:** 1 Department of Dermatology, First Affiliated Hospital of Nanjing Medical University, Nanjing, China; 2 State Key Laboratory of Reproductive Medicine, Nanjing Medical University, Nanjing, China; 3 Department of Immunology, Nanjing Medical University, Nanjing, China; 4 Jiangsu Key Laboratory of Molecular Biology for Skin Diseases and STIs, Institute of Dermatology, Chinese Academy of Medical Sciences &Peking Union Medical College, Nanjing, China; 5 Jiangsu Province Geriatric Hospital, Jiangsu province Geriatric Institute, Nanjing, China

**Keywords:** Meiosis, Immunology

## Abstract

Psoriasis, an immune-mediated inflammatory disease, is associated with poor pregnancy outcomes. Emerging evidence indicates that these defects are likely attributed to compromised oocyte competence. Nevertheless, little is known about the underlying associated mechanisms between psoriasis and poor oocyte quality. In this study, we construct an imiquimod-induced chronic psoriasis-like mouse model to review the effects of psoriasis on oocyte quality. We discover that oocytes from psoriasis-like mice display spindle/chromosome disorganization, kinetochore-microtubule mis-attachment, and aneuploidy. Importantly, our results show that melatonin supplement in vitro and in vivo not only increases the rate of matured oocytes but also significantly attenuates oxidative stress and meiotic defects by restoring mitochondrial function in oocytes from psoriasis-like mice. Altogether, our data uncover the adverse effects of psoriasis symptoms on oocytes, and melatonin supplement ameliorates oxidative stress and meiotic defects of oocytes from psoriatic mice.

## Introduction

Psoriasis is a common immune-mediated inflammatory disease, the prevalence ranges from 0.09 to 11.4% with no difference between male and female [1]. Among females with psoriasis, about half are of reproductive age [1, 2]. Previous research showed that immune-mediated inflammatory diseases including rheumatoid arthritis [3], inflammatory bowel disease [4], systemic lupus erythematosus [5], and Addison’s disease [6] might adversely affect female fertility and pregnancy outcomes, especially during the active period. Female patients with psoriasis have a higher incidence of polycystic ovary syndrome (PCOS) [7] and diminished ovarian reserve [8, 9]. The age-adjusted fertility rate dropped by more than 50% in female with moderate-to-severe psoriasis compared with the general population [10]. An increased risk of spontaneous abortion, preeclampsia, macrosomia, low birth weight, and large for gestational age in female with moderate-to-severe psoriasis has been found out [11–13].

Oocytes are the major determinant of establishing female fertility. Poor oocyte quality can affect embryo development and pregnancy outcomes. Oocytes prepare for fertilization and pass on half of their genes to offspring through two meiosis [14]. Spindle assembly and chromosome movement are two crucial events in meiotic maturation, and any fault in these two processes may result in the production of the abnormal oocyte, such as aneuploid eggs. Aneuploidy plays a crucial role in spontaneous abortion, conception defects, and developmental disorders [15]. The effect of psoriasis on the meiosis of oocytes has not been reported.

Melatonin (N-acetyl-5-methoxy tryptamine), an indoleamine, is generated in a variety of tissues, including the pineal gland, skin [16], and oocyte [17], is a free radical scavenger and natural antioxidant [18]. It has been demonstrated that oxidative stress may be responsible for influencing follicular growth, ovulation, and the quality of oocyte [19]. One of the primary sources of oxidative stress is excessive reactive oxygen species (ROS) produced by the mitochondria [20]. In mice and even in humans, it has been reported that melatonin supplement improves mitochondrial function in germ cells, protecting oocyte from ROS damage and ultimately achieving the full developmental potential of oocytes [21–23]. In addition, melatonin supplement is capable of reducing aneuploidy rate in oocytes from mammal [22, 24, 25]. This evidence shows that a potential relationship between mitochondria, ROS, and melatonin during oocyte development.

In this study, we constructed an imiquimod (IMQ)-induced chronic psoriasis-like mouse model to investigate the effects of psoriasis on oocyte quality and determine whether melatonin treatment improves maternal psoriasis-related oocyte quality.

## Results

### IMQ-induced psoriasis-like mouse model shows systemic chronic inflammation

**Fig. 1:**
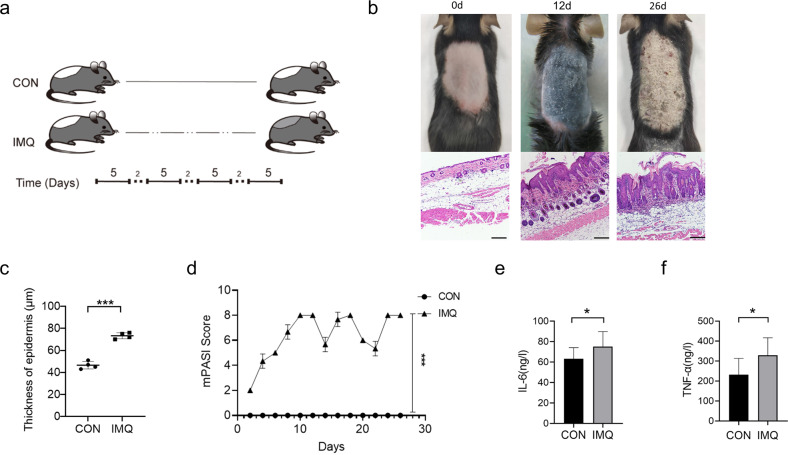
IMQ-induced psoriasis-like mouse model. **a** Schematic diagram of the experimental scheme of IMQ-induced chronic psoriasis-like mice. **b** IMQ treatment effectively induces psoriasis-like lesions (erythema, scales, thickening, and epidermal acanthosis). H&E staining shows that IMQ group has a thickening of epidermal layers, hyperkeratosis, and acanthosis with dermal inflammatory infiltrate. Scale bar: 100 µm. **c** Quantification of epidermal thickness. **d** Cumulative mPASI scores of the back skin were calculated from each group. **e** The serum levels of IL-6 are measured by ELISA (CON *n* = 10, IMQ *n* = 13). **f** The serum levels of TNF-α are measured by ELISA (CON *n* = 8, IMQ *n* = 12). Data are exhibited as mean ± SD. *P < 0.05, ***P < 0.001.

The IMQ-induced mouse model is one of the most classical and widely utilized rodent models in psoriasis research [26]. In order to explore the effect of psoriasis on reproductive function, we established an IMQ-induced chronic psoriasis-like mouse model. Mice were received a topical application of a dose of 50 mg 5% IMQ on the shaved dorsal skin for four 5-day cycles of IMQ with three 2-day recovery period between IMQ cycles (Fig. 1a). The mice treated with IMQ showed erythematous lesions covered with silvery-white squama (Fig. 1b). At day 26, histological analysis of IMQ group shows many histological features of chronic inflammation, including parakeratosis, extended acanthosis, inflammatory cells infiltration (Fig. 1b), and increased epidermal thickness (Fig. 1c). The skin lesion severity was reflected by the modified Psoriasis Area and Severity Index (mPASI) (Fig. 1d).

To explore whether the circulation of IMQ mouse was an inflammatory state, an enzyme-linked immunosorbent assay (ELISA) was performed to detect the levels of interleukin-6 (IL-6) and tumor necrosis factor-α (TNF-α) in serum. Compared with the Control (CON) group, the levels of IL-6 and TNF-α were significantly increased in the IMQ group (mean ± SD of IL-6: 74.93 ± 14.87 ng/L, *n* = 13 vs 63.16 ± 11.00 ng/L, *n* = 10; TNF-α: 329.50 ± 87.00 ng/L, *n* = 12 vs 231.80 ± 81.39 ng/L, *n* = 8; *P* < 0.05) (Fig. 1e, f). These results show that IMQ treatment successfully induces chronic psoriasis-like mouse model and the IMQ mice show inflammatory status.

### Psoriasis-like mice show diminished ovarian reserve

**Fig. 2:**
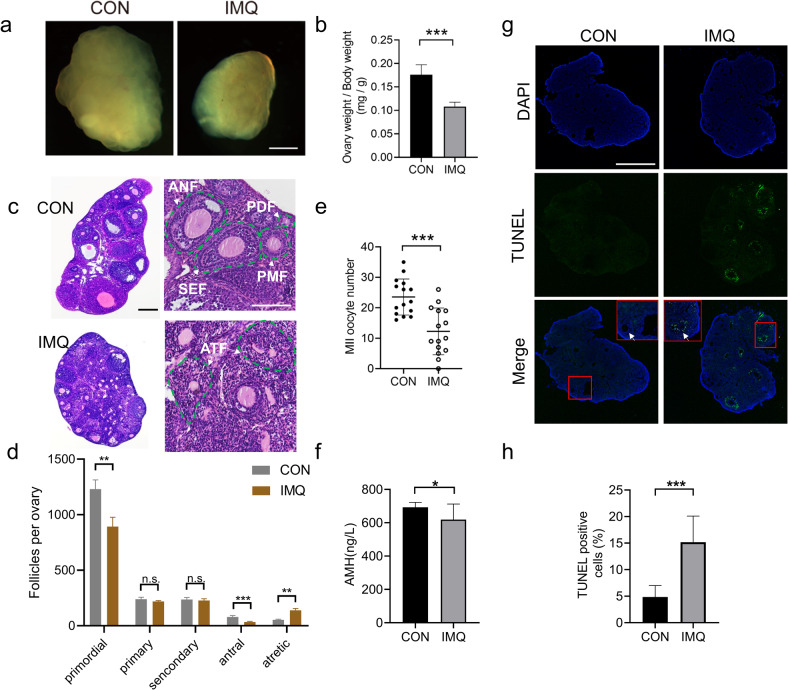
Effects of psoriasis-like symptoms on ovarian reserve. **a** Light microscopy images of ovarian tissues. **b** The ratio of ovarian weight to bodyweight from IMQ and CON groups. **c** Ovarian sections from IMQ and CON group (*n* = 4 for each group). PDF primordial follicles, PMF primary follicles, SEF secondary follicles, ANF antral follicles, ATF atretic follicles. **d** Follicle counts in ovarian sections from IMQ and CON groups. **e** Numbers of MII oocytes obtained from per mouse after superovulation. **f** The serum levels of AMH are measured by ELISA. *n* = 10 for each group. Apoptosis of cells was stained with FITC staining, shown as green fluorescence. The nucleus shown as blue fluorescence with DAPI. **g** Images of ovarian cells after TUNEL staining. White arrow indicates granulosa cells. Apoptotic granulosa cells show a TUNEL-positive signal. **h** Quantitative analysis of TUNEL-positive cells in ovarian tissues. *n* = 6 for each group. Scale bar: 200 µm. Data present as mean ± SD. **P < 0.01, ***P < 0.001, ns none significant.

The ovarian reserve refers to the number and quality of follicle in the ovary, which is a key marker for assessing female fertility [27]. In order to investigate whether and how psoriasis-like symptoms affected female reproductive health, we used ovary sections to evaluate follicular development. IMQ mice had substantially smaller ovaries compared with CON mice (Fig. 2a, b). Follicle-counting results showed that the number of primary and secondary follicles in IMQ and CON ovaries was similar, however, compared with CON group, the number of primordial follicles and antral follicles was considerably decreased (Fig. 2c, d). Accordingly, the number of oocytes obtained from IMQ mice significantly reduced than that from CON mice (12.27 ± 7.69 vs 23.53 ± 5.93, *n* = 15 for each group, *P* < 0.001) (Fig. 2e). Anti-Müllerian hormone (AMH), which belongs to the transforming growth factor-β family and is produced by the granulosa cells of growing follicles, represents a valuable marker of ovarian reserve [28]. Consistent with the follicle-counting results, the IMQ mice showed a significantly declined in serum AMH levels (mean ± SD of AMH: 619.3 ± 93.03 ng/L vs 692.7 ± 29.43 ng/L, *n* = 10 for each group) (Fig. 2f). These results collectively suggested a reduction in ovarian reserve in IMQ mice. Furthermore, morphological staining showed granulosa cells were irregularly arranged. The number of atretic follicles was obviously increased in IMQ ovaries as shown in Fig. 2c, d. Folliculogenesis was impaired as indicated by increased follicular atresia, suggesting diminished ovarian reserve may be due to the defects in folliculogenesis.

The observed folliculogenesis defects in IMQ mice prompted us to examine whether apoptosis had occurred in ovarian cells, particularly in granulosa cells. Ovarian granulosa cells generate necessary nutrients and sex steroids to maintain the development of oocyte [29]. To address this, the TUNEL kit was performed to detect apoptotic cells in the ovary. As shown in Fig. 2g, h, the TUNEL-positive signals were frequently observed in follicular granulosa cells region in IMQ ovaries. The diminished ovarian reserve may be related to increased granulosa cell apoptosis in IMQ ovaries.

### The poor developmental potential and meiotic defects were observed in oocytes from psoriasis-like mice

**Fig. 3:**
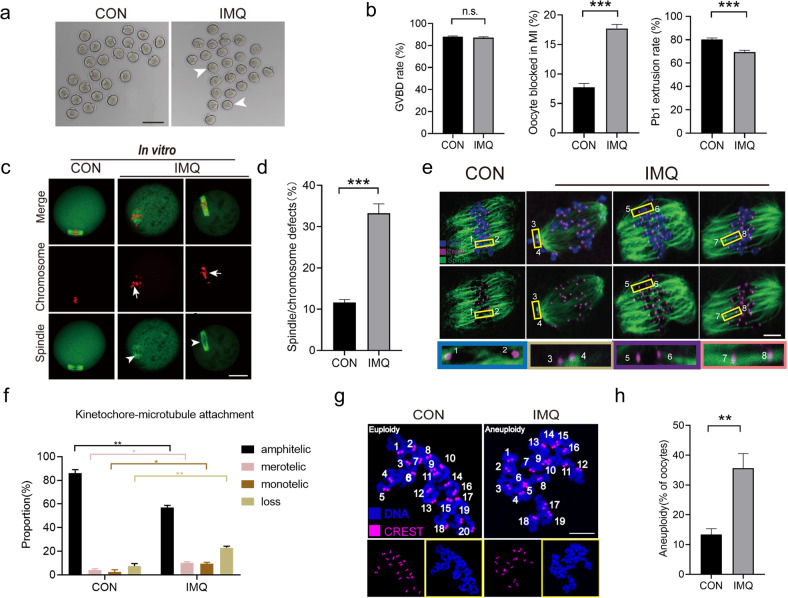
Oocyte maturation and morphological analysis of meiotic apparatus. **a** Representative images of CON and IMQ oocytes. Failure to extrude Pb1 is marked with the white arrowhead. Scale bar: 200 µm. **b** Mensurable analysis of the rate of GVBD, Pb1 extrusion and arrested at MI stage of CON (*n* = 150) and IMQ (*n* = 165) oocytes. In the oocytes from IMQ and CON group, the spindle was stained green by α-tubulin antibody and the chromosomes were counterstained red by PI. **c** Confocal images of spindle morphology and chromosome alignment in the CON and IMQ oocytes that matured in vitro. Arrows point to the disorganized spindles and arrowheads indicate the misaligned chromosomes. Scale bar: 30 µm. **d** Quantitative analysis of 123 CON and 130 IMQ oocytes that matured in vitro with spindle defects and chromosome disalignment. **e** Characteristic confocal images of kinetochore-microtubule attachments are displayed. The kinetochores were dyed purple by CREST antibody. The microtubules were stained green by the anti-tubulin antibody. The chromosomes were dyed blue by Hoechst 33342. Scale bar: 10 µm. Chromosomes labeled 1 and 2 represent examples of amphitelic attachment, chromosomes 3 and 4 represent examples of loss attachment, chromosomes 5 and 6 represent examples of monotelic attachment, chromosomes 7 and 8 represent examples of merotelic attachment. **f** Quantitative analysis of kinetochore-microtubule attachments in CON (*n* = 76) and IMQ (*n* = 88) oocytes. **g** Representative images of karyotype analysis from MII oocytes. Scale bar: 20 µm. **h** The proportion of aneuploidy in CON (*n* = 96) and IMQ (*n* = 121) oocytes. The data were shown as mean ± SD. *P < 0.05, **P < 0.01, ***P < 0.001.

To investigate the developmental potential of IMQ mice oocytes, germinal vesicle (GV) oocytes were cultured in M16 medium. As shown in Fig. 3a, b, the rate of germinal vesicle breakdown (GVBD) had no significant difference after 3 h of culture. Whereas, after 14-h culture, the rate of first polar body (Pb1) extrusion was obviously lower in IMQ oocytes (69.40 ± 1.60%) than that in CON (80.25 ± 1.244%), IMQ oocytes were significantly blocked at metaphase I (MI) stage compared with the CON group (17.73 ± 0.68% vs 7.75 ± 0.62%, *P* < 0.001). In short, these results reveal that maturation progression of oocytes is disturbed in the IMQ group.

It is well-known that an organized spindle is crucial to the accuracy of chromosome segregation [30]. Given this significance, we used immunofluorescence labeling to observe spindle morphology. As shown in Fig. 3c, metaphase II (MII) oocytes in the CON group showed a typical barrel-shaped spindle and well-aligned chromosomes. On the contrary, nearly a third of oocytes collected from the IMQ group displayed disorganized spindle (arrows) or misaligned chromosomes (arrowheads) (Fig. 3c, d).

Kinetochores are multiprotein that interface chromosomes to microtubules of meiotic spindles, have a key role in the remedy of improper microtubule attachments and the coordination of chromosome attachment [31, 32]. The abnormal formation of spindle and the inaccurate arrangement of chromosomes are closely related to kinetochores and microtubules mis-attachment. Hence, we then immunostained the MI oocytes to detect the accuracy of kinetochore-microtubule attachments. As shown in Fig. 3e, f, the great mass of oocytes in the CON group displayed kinetochores were properly attached by microtubules and chromosomes were well-aligned (amphitelic attachment). Nevertheless, the incidence of three mismatches (lost attachment, merotelic attachment, and monotelic attachment) increased significantly in IMQ oocytes compare to CON oocytes.

The defective microtubule–kinetochore attachment would lead to unbalanced pulling forces across kinetochores and spindle, then causing aneuploidy [31]. Therefore, chromosome spreading and kinetochore labeling were used to analyze the karyotype of MII oocytes. As shown in Fig. 3g, h, the aneuploidy was frequently observed in IMQ oocytes compared to CON oocytes (35.68 ± 4.83%, *n* = 101 vs 13.36 ± 1.96%, *n* = 96). Above all, psoriasis-like symptoms disrupt spindle formation and chromosome arrangement in meiosis, consequently increasing the incidence of aneuploidy.

### Psoriasis-like mice show reduced antioxidant capacity

**Fig. 4:**
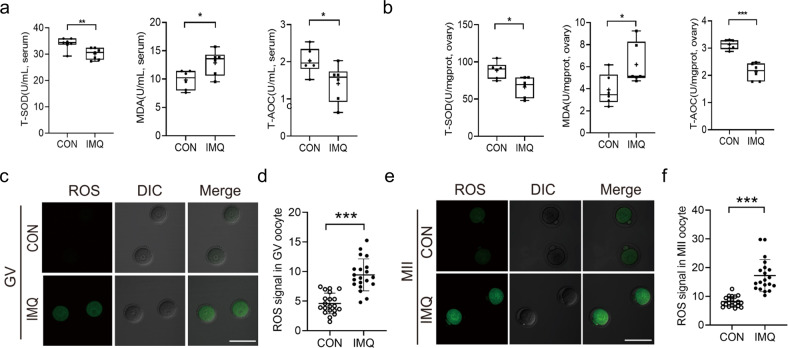
Effects of psoriasis-like symptoms on the serum and ovarian antioxidant capacity ability and oocyte ROS generation. **a** The T-SOD, MDA, and T-AOC levels in the serum from IMQ and CON groups. **b** The T-SOD, MDA, and T-AOC levels in the ovaries from IMQ and CON groups. *n* = 6 for each group. In GV and MII oocytes from CON and IMQ groups, ROS levels were assessed by the intensity of the green fluorescence signal. **c** Confocal images of ROS signal in GV oocytes from CON and IMQ mice. Scale bar: 150 µm. **d** Quantitative analysis of green fluorescence intensity in GV oocytes (*n* = 20 for each group). **e** Confocal images of ROS signal in MII oocytes from CON and IMQ mice. **f** Quantification of the ROS levels in MII oocytes (*n* = 20 for each group). The data are shown as mean ± SD. *P < 0.05, **P < 0.01, ***P < 0.001.

Inflammation and oxidative stress are linked in a self-perpetuating cycle. To evaluate whether the circulation of IMQ mouse is an oxidative stress state, the levels of total superoxide dismutase (T-SOD), malondialdehyde (MDA), and total anti-oxidative capacity (T-AOC) are tested in serum and ovaries. SOD is responsible for converting the superoxide anion into hydrogen peroxide. MDA is a lipid peroxidation marker. The levels of T-SOD and T-AOC were significantly lower in the IMQ group than in the CON group both in serum and ovaries. By comparison, the levels of MDA were obviously increased in the IMQ group than CON group (Fig. 4a, b). The psoriasis-like mice show peroxidation and this peroxidation affects the ovary.

The accumulation of ROS leads to oxidative stress that induces shortening of telomere, microtubule instability, chromosomal segregation disorders, oocyte fragmentation, and fertilization failures [33]. We speculated if the poor oocyte quality in IMQ mice was due to the increased ROS levels. To verify this hypothesis, we decided to utilize CM-H_2_DCFAD to evaluate ROS levels in GV and MII oocytes collected from CON and IMQ mice. Remarkably, quantitation revealed that the ROS signal of IMQ oocytes in both stages was obviously higher than that of CON oocytes (Fig. 4c–f). Our data suggest the accumulated ROS in oocytes may account for the meiotic defects observed in IMQ mice.

### Melatonin supplement during in vitro maturation alleviates meiotic defects in oocytes from psoriasis-like mice

**Fig. 5:**
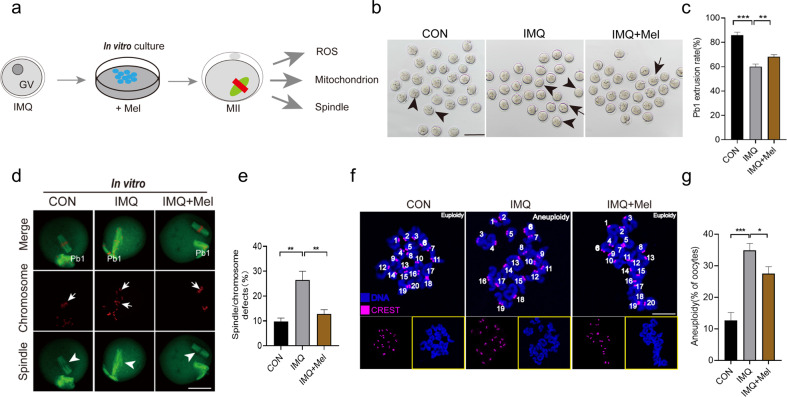
Effects of melatonin supplement during in vitro maturation on the meiotic progression. **a** Diagram of melatonin supplement during in vitro maturation. **b** Phase-contrast images of in vitro-matured oocytes collected from CON, IMQ and IMQ + Mel mice. Scale bars: 200 µm. **c** Mensurable analysis of Pb1 extrusion rate, 150 CON, 165 IMQ, and 140 IMQ + Mel oocytes were included in the experiment. **d** Confocal images of spindle morphology and chromosome alignment in the CON, IMQ, and IMQ + Mel oocytes that matured in vitro. Arrows point to disorganized spindles; arrowheads point to misaligned chromosomes. Scale bar: 40 µm. **e** Quantification of MII oocytes that matured in vitro with spindle defects and chromosome disalignment. 102 CON, 115 IMQ, and 120 IMQ + Mel oocytes were tested. **f** Karyotype analysis diagram of MII oocytes from the CON, IMQ, and IMQ + Mel group. Scale bar: 20 µm. **g** The proportion of aneuploidy in CON (*n* = 20), IMQ (*n* = 25), and IMQ + Mel (*n* = 21) oocytes. The data are shown as mean ± SD. *P < 0.05, **P < 0.01, ***P < 0.001.

As previous reports, melatonin supplement is capable of reducing meiotic defects rate in oocytes from mammals [24, 34], therefore, we further investigated whether melatonin could alleviate oocyte damage caused by psoriasis. GV oocytes were obtained from IMQ mice and then matured in M16 medium supplemented with or without melatonin (Fig. 5a). After in vitro culture for 14 h, quantification of the Pb1 extrusion rate indicated that melatonin promoted oocyte maturation in IMQ mice (Fig. 5b, c). Next, we collected oocytes from CON, IMQ and IMQ mice supplemented with melatonin (IMQ + Mel), which are evaluated the spindle/chromosome organization. Confocal microscopy combined with quantitative analysis indicated that the proportion of defective spindle (arrows) and misaligned chromosomes (arrowheads) in the IMQ group was significantly decreased after melatonin supplement (Fig. 5d, e). Then, we examined the karyotype of MII oocytes through chromosome spreading. Melatonin supplement decreased the aneuploidy rate in IMQ oocytes (Fig. 5f, g). Taken together, in vitro melatonin supplementation partly alleviated the meiotic defects to maintain the euploidy.

### Melatonin supplement attenuates ROS levels and reestablishes mitochondrial function in IMQ oocytes

**Fig. 6:**
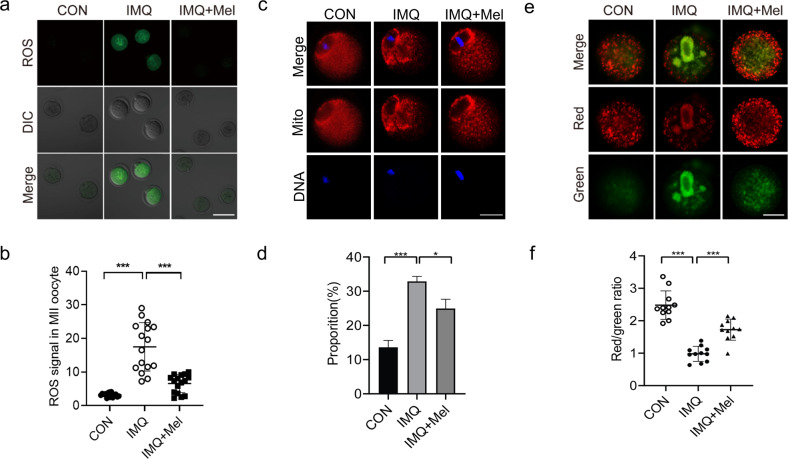
Effects of melatonin supplement during in vitro maturation on ROS generation and mitochondrial function in oocytes from psoriasis-like mice. **a** Confocal images showing ROS signal (green) in CON, IMQ and IMQ + Mel oocytes. Scale bar: 150 µm. **b** Quantitative analysis of ROS signal (*n* = 16 for each group). **c** The images showing red mitochondrial distribution in CON, IMQ and IMQ + Mel oocytes. Scale bar: 30 µm. **d** Quantitative analysis of mitochondrial distribution (*n* = 15 for each group). **e** △Ψm was detected by JC-1 staining in CON, IMQ, and IMQ + Mel oocytes, red signal showed high △Ψm and green signal indicated low △Ψm. Scale bar: 30 µm. **f** Quantitation of JC-1 signal in CON, IMQ, and IMQ + Mel oocytes (*n* = 14 for each group). The data are shown as mean ± SD. *P < 0.05, **P < 0.01, ***P < 0.001.

Next, we asked how melatonin ameliorates the meiotic defects observed in IMQ oocytes. Given the antioxidant properties and mitochondrial protection of melatonin, we chose to examine levels of ROS and mitochondrial function in IMQ oocytes. Fluorescence imaging and intensity estimation showed that melatonin supplementation adequately decreased the accumulation of ROS in IMQ oocytes (Fig. 6a, b). Then, to check the impact of melatonin supplement on mitochondrial function on IMQ oocytes, we examined the mitochondrial distribution by Mito Tracker staining. As shown in Fig. 6c, we observed the accumulated distribution in the fringe of chromosomes and homogenous distribution in the cytoplasm in CON oocytes. Nevertheless, it is noteworthy that more than 33% of mitochondria in IMQ oocytes were absent or clustered in the cytoplasm, and melatonin supplementation diminished this to 24% (Fig. 6d).

We also used JC-1 staining to examine the mitochondrial membrane potential (△Ψm). △Ψm was the driving force for the synthesis of ATP. Red fluorescence presented mitochondria with high △Ψm, while mitochondria with low △Ψm showed green fluorescence (Fig. 6e). We observed that △Ψm of IMQ oocytes was much weaker than that of CON oocytes, but the weakness of IMQ oocytes was greatly improved after melatonin supplementation (Fig. 6e, f). It is worth noting that we proved that H_2_O_2_ treatment increases ROS levels in CON oocytes, which is accompanied by many meiotic defects, such as failed oocyte maturation, mitochondrial inhomogenous distribution, decreased △Ψm, and aberrant spindle/chromosome structure (Supplementary Figs. S1 and 2). Altogether, these results suggest that psoriasis-related oxidative stress in oocytes is improved by melatonin supplement.

### Melatonin administration in vivo relieves oxidative stress and ameliorates meiotic defects in IMQ oocytes

**Fig. 7:**
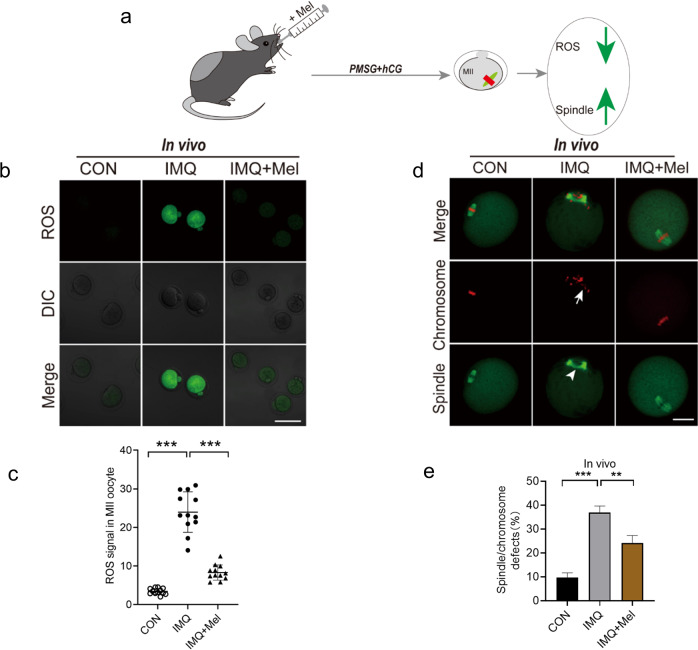
Effects of in vivo administration of melatonin on ROS generation and meiotic apparatus in oocytes that matured in vivo from psoriasis-like mice. **a** Diagram of in vivo administration of melatonin and oocyte that matured in vitro. **b** The images showing ROS fluorescence (green) in CON, IMQ, and IMQ + Mel oocytes. Scale bar: 150 µm. **c** Quantification of ROS signal intensity (*n* = 17 for each group). **d** Confocal images of spindle morphologies and chromosome alignment in the CON, IMQ, and IMQ + Mel oocytes. Scale bar: 30 µm. **e** Quantification of spindle defects and chromosome disalignment in oocytes. CON (*n* = 109), IMQ (*n* = 105), and IMQ + Mel (*n* = 125) oocytes were tested. The data were shown as mean ± SD. **P < 0.01, ***P < 0.001.

**Fig. 8:**
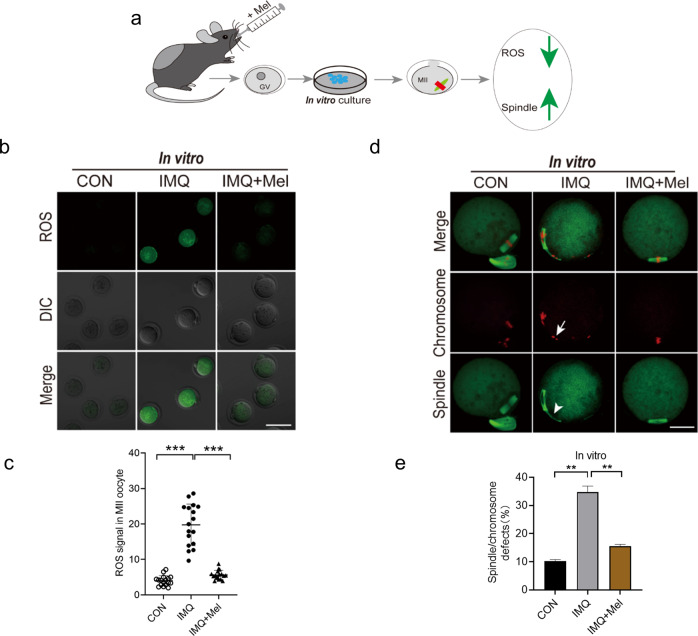
Effects of in vivo administration of melatonin on ROS generation and meiotic apparatus in oocytes that matured in vitro from psoriasis-like mice. **a** Diagram of in vivo administration of melatonin and oocyte that matured in vivo. **b** Images showing ROS signal in CON, IMQ, and IMQ + Mel oocytes. Scale bar: 150 µm. **c** Quantification of ROS fluorescence intensity (*n* = 12 for each group). **d** Confocal images of spindle morphologies and chromosome arrangement in the CON (*n* = 96), IMQ (*n* = 102) and IMQ + Mel (*n* = 98) oocytes that matured in vivo. Scale bar: 30 µm. **e** Quantification of spindle defects and chromosome disalignment in oocytes. The data are shown as mean ± SD. **P < 0.001, ***P < 0.001.

Next, we researched whether in vivo administration of melatonin also could avoid the excessive production of ROS and ameliorate meiotic defects in IMQ mice. To test this hypothesis, we performed a melatonin gavage experiment. Oocytes from IMQ mice intragastric with melatonin were conducted in vitro and vivo maturation (IMQ + Mel). Then, we evaluated ROS levels and spindle organization. Unsurprisingly, quantitative analysis revealed that most oocytes obtained from IMQ + Mel mice reduced the levels of ROS and spindle defects. This result was consistent with adding melatonin to a maturation medium (Figs. 7 and 8). Collectively, these data indicate that melatonin treatment (both in vivo administration and in vitro supplementation) partly ameliorates meiotic defects by reducing oxidative stress in oocytes from IMQ mice, consequently improving oocyte quality. The melatonin treatment in vivo had no significant effects on the skin lesions of IMQ-induced psoriatic mice.

## Discussion

Psoriasis usually presents in a bimodal age distribution, and the earlier peak incidence is from 20 to 40 years old [1]. In early-onset psoriasis (beginning before age 40 years), it is often associated with HLA-Cw6 [35]. Emerging evidence indicates that HLA-C takes part in the pathogenesis of unexplained consecutive recurrent miscarriage [36]. Genetic factors may also contribute to poor pregnancy outcomes in patients with psoriasis.

Chronic inflammatory conditions are connected with unregulated and persistent chemokine and cytokine synthesis secretion, accompanied by the activation of harmful signal transduction [37]. TNF-α and IL-6, these pro-inflammatory cytokines activate the Toll-like receptor (TLR)4/TLR2/nuclear factor-κB signal transduction pathway and transcription 3 signaling pathway to produce more pro-inflammatory cytokines [38]. In our study, serum levels of lL-6 and TNF-α from psoriasis-like mice were elevated. When chemokine and cytokines were released into the circulation, they might induce inflammatory responses in other tissues and organs including the ovary and had an adverse effect on ovarian folliculogenesis and ovulation [39]. These cytokines seemed to play a curial role in the regulation of the development and atresia of follicles [40]. We found that the atretic follicles of psoriasis-like mice were increased, primary follicles were reduced and the number of ovulations was decreased. Meanwhile, the serum AMH level which is a marker of mammalian ovarian reserve showed a significant decrease in psoriasis-like mice compared with CON mice. Reduced ovarian reserve in psoriasis-like mice was consistent with previous studies in a population with psoriasis [8, 9].

Chronic inflammation and ROS are interdependent, each to be the target of the other. During inflammation, inflammatory cells are recruited to the site of injury, which results in a “respiratory burst” due to increased uptake of oxygen [41]. The increase of inflammatory cytokines can stimulate the production of free radicals, further aggravating oxidative stress [42]. In line with our observations, the T-SOD, MDA, and T-AOC levels in serum and ovaries from psoriasis-like mice associated with antioxidant capacity, showed oxidative stress status in tissues.

Primordial follicles were composed of a primary oocyte that is blocked in meiotic diplotene and a layer of flattened granulosa cells surrounding it. As granulosa cell proliferation and fluid accumulation, primordial follicles gradually evolve into primary, secondary, and tertiary follicles [43]. The visible sign of primordial follicle is being recruited is the gradual change of granulosa cells from a squamous to a cuboidal shape. As reported, oxidative stress and granulosa cell apoptosis play a key role in the development of follicular atresia [44]. Our study showed that the level of oxidative stress and the number of apoptotic granulosa cells and atretic follicles in psoriasis-like mice was obviously increased compared with the CON mice.

Accumulation of ROS in the follicular fluid deteriorates oocyte quality and pregnancy outcome, there are growing reports on ROS which plays a role in the female with preeclampsia [45], birth defects [46], abortion [47], infertility [48], and other diseases. In our experiment, we tested the significantly increased ROS levels in oocytes (both GV and MII) from psoriasis-like mice. In line with this, a high incidence of spindle/chromosomes disorganization was observed in oocytes from psoriasis-like mice. Spindle disorganization is always accompanied by the incorrect interaction between kinetochores and microtubules, causing an unbalanced pull in chromosome segregation, and this eventually leads to the increased aneuploidy rate [31, 49]. These results were consistent with our experimental data on the high frequency of defective kinetochore-microtubule attachment and aneuploidy. To sum up, one of the causes of the meiotic defects in oocytes from psoriasis-like mice is superfluous ROS production.

Melatonin is the primary neuroendocrine secretion of the pineal gland, which can regulate the circadian rhythms and is also a robust antioxidant [18]. In this study, our aim is to find out whether melatonin supplementation can reduce ROS levels and improve the poor quality of IMQ oocytes. Our findings validate that melatonin supplementation partly increased the maturation rate of IMQ oocytes by promoting nuclear and cytoplasmic maturation.

Mitochondria are crucial animal organelle. Optimum energy production is required for oocyte maturation, fertilization, and embryo development [50]. Growing evidence shows that the abnormal mitochondria influence energy production, lead to the redundant accumulation of ROS, result in spindle/chromosome abnormalities, aneuploidy, and affect the developmental potentiality of the oocytes [51–53]. Mitochondrial uniform distribution in the cytoplasm is identified as the marker of oocytes with higher developmental potential [54]. △Ψm is an indicator of mitochondrial activity and is responsible for energy production, redox balance, signaling, and metabolism [55]. Uneven distribution patterns of mitochondria and weaker △Ψm were observed in oocytes from psoriasis-like mice and melatonin treatment could prevent this abnormality. At the same time, melatonin treatment markedly decreased the ROS levels in oocytes from psoriasis-like mice, thereby restoring the spindle/chromosome structural defects and reducing the frequency of aneuploidy. We provide evidence documenting that melatonin treatment restores mitochondrial function of oocytes to suppress the accumulation of ROS and improves the quality of oocytes from psoriasis-like mice.

Collectively, our study was the first to construct chronic psoriasis-like mouse model to examine the effects of maternal psoriasis on oocyte quality, which revealed that the psoriasis state damages the oocyte quality and ovarian reserve. Psoriasis causes oocyte mitochondrial dysfunction and oxidative stress, and brings about meiotic defects. In addition, we found that melatonin supplement not only increases the rate of matured oocytes but also significantly attenuates oxidative stress and meiotic defects by restoring mitochondrial function in oocytes from psoriasis-like mice. Whether melatonin or other treatment can improve psoriasis-related decline in ovarian reserve, requires further exploration.

## Materials and methods

### Chronic psoriasis-like mouse model

Six to seven-week female C57BL/6 mice were used in our studies. All animal experiments were conducted in accordance with relevant ethical guidelines and codes stipulated by the Animal Care and Use Committee of Nanjing Medical University.

Psoriasis-like mice were induced by 5% IMQ cream (Aldara, 3 M Health Care Limited, UK). Mice received a topical application of 50 mg 5% IMQ on the shaved dorsal skin for four 5-day cycles of IMQ with three 2-day recovery period between IMQ cycles. For convenience, the IMQ-treated mice were named “IMQ mice” and the untreated mice were named “CON mice”. The skin was examined every day, and the dorsa were photographed. The mice were sacrificed by cervical dislocation on day 26, and their skin, serum, ovaries, and oocytes were collected for the next experiment.

### H&E staining and histological analysis

For histological examination, mice skin and ovaries were fixed in 10% paraformaldehyde and then dehydrated in an alcohol gradient at room temperature. For serial slicing, the tissues were embedded in paraffin. After serial slices with a thickness of 5 μm were produced, stained with hematoxylin (31890610, Sigma, USA) and eosin (HHS16, Sigma, USA).

### ELISA

Mouse plasma was centrifuged at 2500 × *g* for 15 min at 4 °C to layer, and the upper serum was stored at −80 °C for later ELISA. Used IL-6 (H007) and TNF-α (H052) ELISA kits (JIANCHENG, China) to test the concentrations of IL-6 and TNF-α in conformity to the instructions. The levels of AMH (H324) were assessed according to the manufacturer’s protocols of the ELISA kit (JIANCHENG, China).

### Assessment of cells apoptosis in ovary

Apoptosis of ovarian cells was detected by TdT-mediated dUTP nick-end labeling (TUNEL) staining. In brief, ovaries were fixed in 4% paraformaldehyde, dehydrated with 30% sucrose solution, embedded in optimal cutting temperature compound (OCT), and then cut into 10μm sections at −20 °C. Ovarian frozen sections were stained with the TUNEL kit (G1501, Servicebio, China) according to the manufacturer’s guides, observed, and photographed by a Nikon fluorescence microscope.

### The experiment of T-SOD, MDA, and T-AOC of serum and ovaries

The levels of T-SOD (A001), MDA (A003), and T-AOC (A015) in mouse serum and ovaries were detected by assay kits (JIANCHENG, China). The operation was carried out in accordance with the manufacturer’s instructions.

### Antibodies

In this study, we used the following antibodies: mouse monoclonal FITC-conjugated anti-α-tubulin antibodies (F2168, Sigma, USA; 1:500); human anti-centromere CREST antibody (15234, Antibodies Incorporated, USA; 1:500); Cy5-conjugated donkey anti-human IgG (709605149, Jackson ImmunoResearch Laboratory, USA; 1:500).

### Melatonin treatment

The 6–7-week female C57BL/6 mice were randomly assigned to three groups: CON group, IMQ group, and IMQ + melatonin (IMQ + Mel) group. In our study, approximately 150 CON mice, 200 IMQ mice, 100 IMQ + Mel mice were sacrificed. For in vivo experiment, the day before IMQ application mice were given melatonin (M5250, Sigma, USA) of daily oral doses of 30 mg/kg bodyweight for 27 days. The dosage of melatonin depended on the published literature [24, 34] and our preliminary sifting. For in vitro supplementation, we chose M16 medium containing 1 μmol/L of melatonin to culture GV oocytes from psoriasis-like mice according to the published literature [24, 25, 34, 56].

### Oocyte collection and culture

Female mice were received an intraperitoneal injection of 5 IU Pregnant Mares Serum Gonadotropin (PMSG) for superovulation. After 2 days, antral follicles were artificially ruptured to obtain cumulus-oocyte complexes (COCs). Subsequently, cumulus cells were removed by repeated pipetting. GV oocytes were cultured further in M16 medium covered with mineral oil at 37 °C in an atmosphere of 5% CO_2_ incubator. To retrieve MII oocytes, mice that received intraperitoneal injection of 5 IU PMSG before 48 h were intraperitoneally injected with 5 IU human Chorionic Gonadotropin (hCG), then sacrificed 14 h after hCG priming. COCs were obtained from the oviduct ampullae, cumulus cells were removed in a medium containing 0.5 mg/mL hyaluronidase at 37 °C.

### Immunofluorescence and confocal microscopy

Immunofluorescence and confocal microscopy were conducted based on previous research reports [57]. To fixation, oocytes were placed in 4% PFA at room temperature for 30 min. To permeabilization, oocytes were placed in 0.5% Triton X-100 for 18 min. After blocking in PBS supplemented with 1% BSA for 1 h, oocytes were dyed with anti-α-tubulin-FITC antibody to visualize the spindle. To visualize kinetochores, oocytes were stained with CREST and secondary antibody for 1 h. Then, to visualize chromosomes, oocytes were co-labeled with Hoechst 33342 or PI for 10 min. At last, oocytes were transferred to microscope slides with antifade medium (Vectashield, USA) and timely examined under a Laser Scanning Confocal Microscope (LSM 800, Zeiss, Germany).

### Chromosome spread

Aiming at getting rid of zona pellucida, MII oocytes were placed in Tyrode’s buffer (pH 2.5) momently (about 40 s) at room temperature. The samples were transferred to M2 medium for 10 min to recovery, subsequently, fixed in 1% paraformaldehyde with 0.15% Triton X-100. Following kinetochore staining by CREST and Cy5-conjugated secondary antibody and chromosomes incubating with Hoechst 33342, the karyotype was examined by Laser Scanning Confocal Microscope.

### ROS measurement

In order to detect the levels of ROS generation, CM-H_2_DCFDA (C6827, Life Technologies, USA) was used in this study. Oocytes were incubated in M2 medium containing 5 µmol/L CM-H_2_DCFDA for 30 min at 37 °C in a 5% CO_2_ incubator and placed on Nunc™ Glass Bottom Dishes (15082, Thermo Scientific, USA) after being washed three times. The CM-H_2_DCFDA signal of oocytes was detected promptly by the Zeiss LSM 700 META confocal system.

### Mitochondrial distribution analyses and △Ψm measurement

For active mitochondrial distribution analysis, oocytes were incubated in M16 medium containing 200 nM Mito Tracker Red (Molecular Probes, USA) for 30 min in the dark. After DAPI counterstaining, samples were mounted on Nunc™ Glass Bottom Dishes and examined promptly by the Zeiss LSM 700 META confocal system.

Using Mito Probe JC-1 Assay Kit (M34152, Thermo Fisher Scientific, USA) evaluated△Ψm. Oocytes were incubated in M16 medium containing 2 μM JC-1 for 30 min in the dark. After being washed three times for 10 min each, samples were placed on Nunc™ Glass Bottom Dishes and examined promptly by the Zeiss LSM 700 META confocal system.

### Statistical analysis

All percentages or values from at least three replicate experiments were expressed as mean ± SD. Data were analyzed by GraphPad Prism 8. Statistical comparisons were made by Student’s *t* test. An electronic laboratory notebook was not used. *P* < 0.05 was considered to be statistically significant.

## Supplementary information

Figure S1

Figure S2

Supplementary figure legends

A reproducibility checklist

## Data Availability

The data used and analyzed during this study are available from the corresponding author on reasonable request.
