# Supplementary material for: Oxidative stress induces meiotic defects of oocytes in a mouse psoriasis model
Source: Cell Death Dis. 2022 May 19;13(5):474. doi: 10.1038/s41419-022-04948-w (PMC9119936; doi:10.1038/s41419-022-04948-w)
Supplement: Supplementary file 3 — Supplementary figure legends [file 41419_2022_4948_MOESM3_ESM.docx]

**Figure S1. Effects of H_2_O_2_ treatment on meiotic progression in oocytes. a** Phase-contrast images of oocytes that matured in vitro with and without H_2_O_2_ treatment. Oocytes were treated with 10 μM H_2_O_2_. Scale bars: 200 µm. **b** Graph showing the Pb1 extrusion rate in CON (n=60) and H_2_O_2_-treated (n=71) oocytes. **c** Confocal images of spindle morphologies and chromosome alignment in CON and H_2_O_2_-treated oocytes. Scale bar: 40 µm **d** Quantification of CON (n=78) and H_2_O_2_-treated (n=86) oocytes. The data were shown as mean ± SD. ***P <0.001.

**Figure S2. Effects of H_2_O_2_ treatment on ROS and mitochondrial function in oocytes. a** Images showing ROS signal in CON and H_2_O_2_-treated oocytes. Scale bar: 250 µm. **b** Quantitative analysis of ROS fluorescence intensity (n=10) and H_2_O_2_-treated (n=15) oocytes. **c** Typical images of mitochondrial distribution in CON and H_2_O_2_-treated oocytes. Scale bar, 30 µm. **d** Histogram showing the abnormal rate of mitochondrial distribution in CON (n=17) and H_2_O_2_-treated (n=19) oocytes. **e** Typical images of △Ψm in CON and H_2_O_2_-treated oocytes. Scale bar: 30 µm. **f** Quantitation of JC-1 signal in CON (n=16) and H_2_O_2_-treated (n=17) oocytes. The data were shown as mean ± SD. ***P <0.001.
